# Ivacaftor and symptoms of extra-oesophageal reflux in patients with cystic fibrosis and *G551D* mutation

**DOI:** 10.1016/j.jcf.2016.07.004

**Published:** 2017-01

**Authors:** Gemma L. Zeybel, Jeffrey P. Pearson, Amaran Krishnan, Stephen J. Bourke, Simon Doe, Alan Anderson, Shoaib Faruqi, Alyn H. Morice, Rhys Jones, Melissa McDonnell, Mujdat Zeybel, Peter W. Dettmar, Malcolm Brodlie, Chris Ward

**Affiliations:** a Institute for Cell and Molecular Bioscience, Newcastle University, Catherine Cookson Building, Framlington Place, Newcastle Upon Tyne NE2 4HH, United Kingdom; b Institute for Cellular Medicine, Medical School, Newcastle University, Catherine Cookson Building, Framlington Place, Newcastle Upon Tyne NE2 4HH, United Kingdom; c Northern Aerodigestive group, Royal Victoria Infirmary, Newcastle upon Tyne NE1 4LP, United Kingdom; d Department of Respiratory Medicine, Royal Victoria Infirmary, Newcastle upon Tyne NE1 4LP, United Kingdom; e Academic Department of Respiratory Medicine, Hull York Medical School, University of Hull, Castle Hill Hospital, Cottingham, United Kingdom; f School of Medicine, Koç University, Istanbul, Turkey; g RD Biomed Ltd, Castle Hill Hospital, Cottingham, United Kingdom; h Department of Paediatric Respiratory Medicine, Great North Children's Hospital, Queen Victoria Road, Newcastle upon Tyne NE1 4LP, United Kingdom

**Keywords:** G551D, CFTR potentiator, Gastrointestinal, Real life data

## Abstract

**Background:**

Extra-oesophageal reflux (EOR) may lead to microaspiration in patients with cystic fibrosis (CF), a probable cause of deteriorating lung function. Successful clinical trials of ivacaftor highlight opportunities to understand EOR in a real world study.

**Methods:**

Data from 12 patients with CF and the *G551D* mutation prescribed ivacaftor (150 mg bd) was collected at baseline, 6, 26 and 52 weeks. The changes in symptoms of EOR were assessed by questionnaire (reflux symptom index (RSI) and Hull airway reflux questionnaire (HARQ)).

**Results:**

Six patients presented EOR at baseline (RSI > 13; median 13; range 2–29) and 5 presented airway reflux (HARQ > 13; median 12; range 3 to 33). Treatment with ivacaftor was associated with a significant reduction of EOR symptoms (P < 0 ∙ 04 versus baseline) denoted by the reflux symptom index and Hull airway reflux questionnaire.

**Conclusion:**

Ivacaftor treatment was beneficial for patients with symptoms of EOR, thought to be a precursor to microaspiration.

## 1 Introduction

Cystic fibrosis (CF) is a genetic condition caused by abnormalities in the CF transmembrane conductance regulator (*CFTR*) gene. Patients with CF experience life-long morbidity and premature mortality, associated with lung disease. The exact pathophysiology of CF remains an area of much interest and research. It is recognised as a multi-system disorder involving the respiratory and gastrointestinal tract [1], [2], [3]. Gastrointestinal problems constitute a significant consideration in patients with CF. Extra-oesophageal reflux (EOR) is known to occur frequently in the adult CF population, the prevalence ranges from 55 to 90% in different studies [4]. EOR is the retrograde movement of reflux which leaves the oesophagus, leading to symptoms such as cough, hoarseness, post-nasal drip and globus [10]. Reflux-induced cough and reported EOR symptoms have been associated with reduced lung function [4]. In pilot work we detected bile acids in the lower airways of 9 patients homozygous for *F508del* with advanced CF lung disease at the time of lung transplantation [5], and this potential source of injury persisted after lung transplantation [6]. This indicated that microaspiration of refluxate in patients with CF might be a mechanism of injury linking gastrointestinal problems with lung disease [7].

The clinical success associated with trials of the CFTR potentiator ivacaftor (Vertex Pharmaceuticals, Massachusetts, USA) in patients with CF and the *G551D* mutation [8] highlights an opportunity for pragmatic studies to understand clinically relevant CF pathophysiology and symptoms [2]. It is recognised that apart from formal trial populations, data from clinical populations, gained in the post-approval era of ivacaftor is highly valued [2], [9]. Rowe and colleagues provided an excellent review of the North American experience of ivacaftor treatment in patients with CF and the *G551D* mutation [3]. They confirmed previous data observing gains in lung function after 1 month of ivacaftor treatment and weight gain accompanied by body mass index (BMI) after 6 months of treatment. In particular, a sub-study of seven patients underwent postprandial intestinal pH measurements to investigate the role of ivacaftor within intestinal pH profiles. Following 1 month of ivacaftor treatment, a significant improvement was observed in the early ability to promote postprandial duodenal neutralisation when compared to baseline measurements. The observed improvement of weight gain, occurring with ivacaftor treatment, along with the four week longitudinal intestinal pH data highlights a potentially central role for CFTR in gastrointestinal complications modified by this new approach to therapy [3].

With longitudinal gastrointestinal data rare in the specialised setting of ivacaftor treatment, and absent beyond the four week time point, we performed an uncontrolled, observational study of twelve patients with CF with one copy of the *G551D CFTR* mutation. Patients were treated with ivacaftor for 12 months. We evaluated validated questionnaire based instruments of EOR, selected prospectively following a formal literature review and hypothesised that clinical improvements in lung function associated with ivacaftor therapy would be associated with a positive and sustained impact on symptoms of reflux.

## 2 Methods

Ethical approval was granted by County Durham & Tees Valley 2 Research Ethics Committee (REC NO: 10/H0908/8). The ethics complied with the principles laid down in the Declaration of Helsinki. Clinic patients receiving ivacaftor (150 mg bd) for the first time were invited to take part in this study.

A total of twelve patients with one copy of the *G551D CFTR* mutation were recruited (six female, median age 24 years, Table 1) to the study from the Regional Adult CF Service, Royal Victoria Infirmary, Newcastle upon Tyne. We obtained written informed consent from each patient during recruitment.

Following directions from the NHS guidelines, sweat chloride levels were assessed at baseline to assess eligibility for the treatment programme (a baseline sweat chloride concentration of > 60 mmol/L was required for treatment to observe a > 30% fall following ivacaftor). Seven patients were prescribed a Proton Pump Inhibitor (PPI) and one Histamine_2_-Receptor Agonist (H_2_RA) prior to starting ivacaftor. There were six patients prescribed with azithromycin, two patients were taking a 250 mg dose every day and four patients were taking 500 mg three times per week. Of the total number of patients, 11 were pancreatic insufficient and thus prescribed pancreatic enzyme replacement therapy (PERT) (CREON® range 2500 to 7700 units/kg/day). These medications continued throughout the study.

Validated questionnaires designed to assess the symptoms of extra-oesophageal reflux disease were selected following a formal review of the literature (RJ, MM, GZ). This led us to a prospective decision to adopt the Hull airway reflux questionnaire (HARQ) and reflux symptom index (RSI) score. These questionnaires have previously been validated against gold standard pH impedance measurements. In brief, the RSI is a patient reported outcome measure designed to assess laryngeal symptoms secondary to reflux. [10] The HARQ was designed by respiratory physicians to assess the impact of reflux on the airways and was validated amongst 185 patients and 70 volunteers [11]. The two validated reflux questionnaires were completed by the patients, on initial assessment for receiving ivacaftor (baseline), six, 26 and 52 weeks after first taking the medication. The RSI questionnaire assessed EOR symptoms (max score 45, score < 12 classed as not EOR symptomatic) [10]. The HARQ was used to assess cough related EOR (max score 70, score < 12 classed as not being cough related EOR symptomatic) [11].

Measurements of sweat chloride (mmol/L), weight (kg), and pulmonary function (forced expiratory volume in 1 s (FEV_1_) and forced vital capacity (FVC) percentage of predicted) were collected to assess clinical efficiency in line with the North of England specialised Commissioning Group and the NHS commissioning policy (NHSCB/A01/P/b). These parameters were recorded at baseline, six, 26, and except for sweat chloride, 52 weeks. The predicted values for pulmonary function measurements were corrected according to age, gender, and height reference ranges using European standard reference equations [12].

GraphPad Prism version six (GraphPad Software Inc., La Jolla, California, USA) and MiniTab version 16 (MiniTab Inc., State College, PA, USA) were used for statistical analysis. We performed nonparametric tests due to the small number of patients, these tests do not assume any distribution for the data, which are expressed as median and range. The one-sample Wilcoxon signed rank test was performed on all 12 patients to compare the median difference of each follow-up group. The Mann–Whitney test was used to compare unpaired data. The Friedman test was used for one-way repeated measures analysis. Conventional levels of statistical significance testing were used with P ≤ 0.05 considered significant.

## 3 Results

Data was available from 12 patients at baseline, and following six, 26, and 52 weeks of ivacaftor treatment. The reflux questionnaire data was missing for one patient at 52 weeks due to the patient relocating to another country.

### 3.1 Extra-oesophageal reflux

**Fig. 1:**
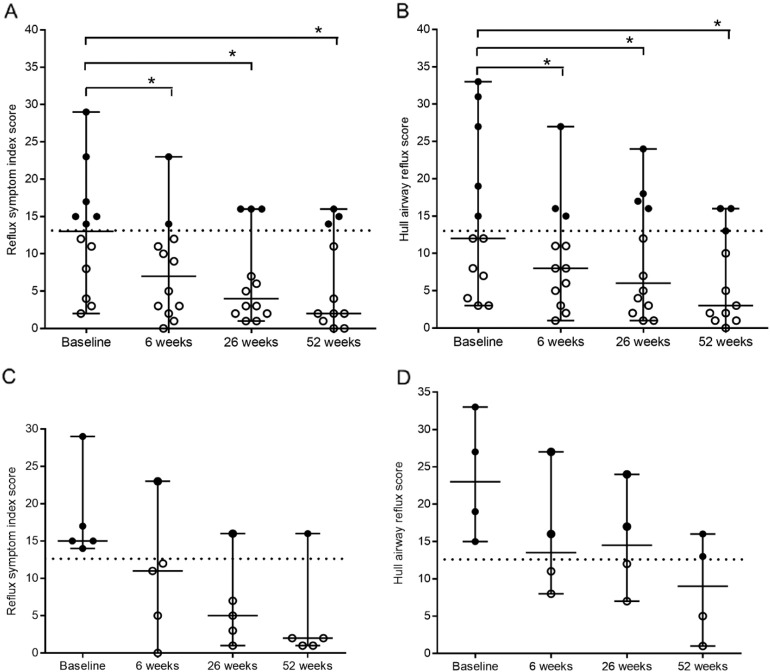
Median scores of extra-oesophageal reflux symptom severity from baseline to 52 weeks with ivacaftor treatment (150 mg bd) at 6, 26, and 52 weeks *P < 0.05. (A) The reflux symptom index score at baseline was 13 (range 2 to 29). It reduced to a median score of 7 at 6 weeks (range 0 to 23; P = 0.0054); 4 at 26 weeks (range 1 to 16; P = 0.0054) and 2 at 52 weeks (range 0 to 16; P = 0.02). (B) The Hull airway reflux questionnaire score at baseline was 12 (range 3 to 33). It reduced to a median score of 8 (range 1 to 27; P = 0.02) at 6 weeks; 6 (range 1 to 24; P = 0.01) at 26 weeks and 3 at 52 weeks (range 0 to 16; P = 0.04). (C) Five patients were EOR symptomatic determined by the reflux symptom index score (RSI > 13) on PPI/H2RA at baseline. Of these patients, 4 became free of symptoms at during the remainder of the study. (D) EOR/cough determined by the Hull airway reflux questionnaire (HARQ > 13) identified 4 symptomatic patients at baseline on PPI/H2RA. This reduced to 2 symptomatic patients during the remainder of the study. Note: bd twice daily; PPI proton pump inhibitors; H2RA Histamine2 receptor agonists; EOR extra-oesophageal reflux.

For the patient group as a whole (n = 12), the median RSI score at baseline was 13 (range 2 to 29). There was a significant decrease in EOR symptoms at the 6 weeks (median 7; range 0 to 23; p = 0.005 versus baseline), 26 weeks (median 4; range 1 to 16; P = 0.005 versus baseline) and 52 weeks (n = 11; median 2; range 0 to 16; P = 0.02) follow-up (Friedman test n = 11 P = 0.03). EOR symptoms were present in six patients at baseline (RSI score > 13). Four of these patients became non-symptomatic (RSI score < 12) throughout the treatment of ivacaftor (Fig. 1A).

Of the total 12 patients, the median HARQ score at baseline was 12 (range 3 to 33). There was a significant decrease in cough related EOR at 6 weeks (median 8; range 1 to 27; P = 0.003 versus baseline), 26 weeks (median 6; range 1 to 24; P = 0 ⋅ 01 versus baseline) and 52 weeks (n = 11; median 3; range 0 to 16; P = 0 ⋅ 04 versus baseline) follow-up. Five patients were positive for cough related EOR symptoms at baseline (HARQ score > 13) (Friedman test n = 11 P = 0.006). During ivacaftor treatment the number of symptomatic patients decreased to three patients at 6 weeks, four patients after 26 weeks and three patients after 52 weeks (Fig. 1B).

### 3.2 Symptoms of reflux in patients on PPI medication

Of the total 12 patients, 9 were treated with acid suppressive therapy. Symptoms of EOR remained in those patients treated with PPI therapy and H2RA's at baseline. Five of these patients were symptomatic determined by the RSI at baseline, with 4 determined by the HARQ at baseline. Symptom severity decreased in all these patients during ivacaftor treatment. There were four patients with an RSI score below 12 and 2 with a HARQ score below 12, classified as non-symptomatic at the 6 week, 26 week and 52 week follow-up. There were no discernible differences in the effect of ivacaftor treatment associated with reflux medication (Fig. 1C and D).

### 3.3 Lung function

**Fig. 2:**
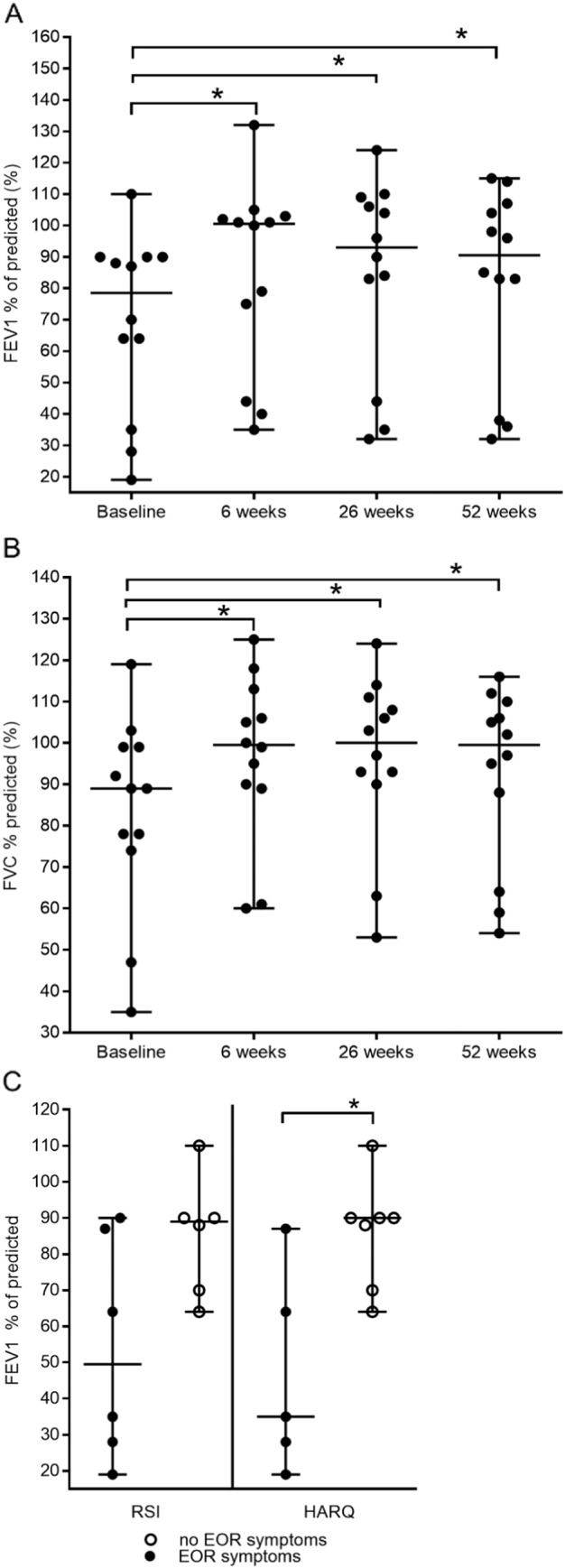
(A) median baseline percentage of predicted FEV1 was 79% (range 19 to 110). Increasing from baseline to 101% (range 35 to 132; P = 0.0005) at 6 weeks; 93% (range 32 to 124; P = 0.001) at 26 weeks; 91 (range 32 to 115; P = 0.005) at 52 weeks. (B) Median baseline percentage predicted of FVC was 89% (range 35 to 119). Increasing to 100% (range 60 to 125; P = 0.0005) at 6 weeks; 100% (range 53 to 124; P = 0.0005) at 26 weeks and 100% (range 54 to 116; P = 0.05) at 52 weeks. (C) Comparison of FEV1 % predicted and the presence of extra-oesophageal reflux (EOR) symptoms at baseline. Lung function analysed from baseline over a time frame of 52-week treatment with ivacaftor (150 mg bd) at a follow-up period of 6, 26 weeks and 52 weeks. (A) Patients who were EOR symptomatic had a lower FEV1 predicted at baseline (reflux symptom index median 50%; range 19 to 90; Hull airway reflux median 35%; range 19 to 87) compared to patients with no symptoms (reflux symptom index median 89%; range 64 to 110; Hull airway reflux median 90%; range 64 to 110; P = 0.01). Note: bd (twice daily); FEV1 (forced expiratory volume in 1 s); FVC (Forced vital capacity). RSI reflux symptom index; HARQ Hull airway reflux questionnaire; EOR extra-oesophageal reflux.

For the whole study group, the median FEV_1_ predicted significantly increased from 79% (range 19 to 110) at baseline to 101% (range 35 to 132) at the six week follow-up (P = 0 ⋅ 0005 versus baseline). The FEV_1_ predicted remained significantly increased from baseline at 93% (range 32 to 124) during the 26 week follow-up (P = 0 ⋅ 001 versus baseline) and 91% (range 32 to 115) during the 52 week follow-up (P = 0.005 versus baseline) (Friedman test n = 12 P = 0.0003) (Fig. 2A).

The median FVC % predicted was 89% at baseline (range 35 to 119). The FVC % predicted significantly increased to a median of 100% (range 60 to 125; P = 0 ⋅ 0005 versus baseline) at the six week follow-up, 100% (range 53 to 124; P = 0 ⋅ 0005 versus baseline) at 26 weeks and to 100% (range 54 to 116; P = 0 ⋅ 05 versus baseline) at 52 weeks. (Friedman test n = 12, P = 0.0003) (Fig. 2 B).

### 3.4 Lung function and symptoms of EOR at baseline

Of the total 12 patients, the median FEV1 predicted was 79% (range 19 to 110) at baseline (Fig. 2B). At this time, the median lung function of EOR symptomatic patients determined by the RSI was 50% (range 19 and 90) and 35% determined by the HARQ (range 19 to 87). The median FEV1 predicted of the group of patients who did not present EOR symptoms at baseline was significantly higher when compared to the EOR symptomatic group (89% denoted by the RSI; range 64 to 110 and 90% denoted by the HARQ; range 64 to 110; P = 0 ∙ 01) (Fig. 2C).

### 3.5 Sweat chloride

**Fig. 3:**
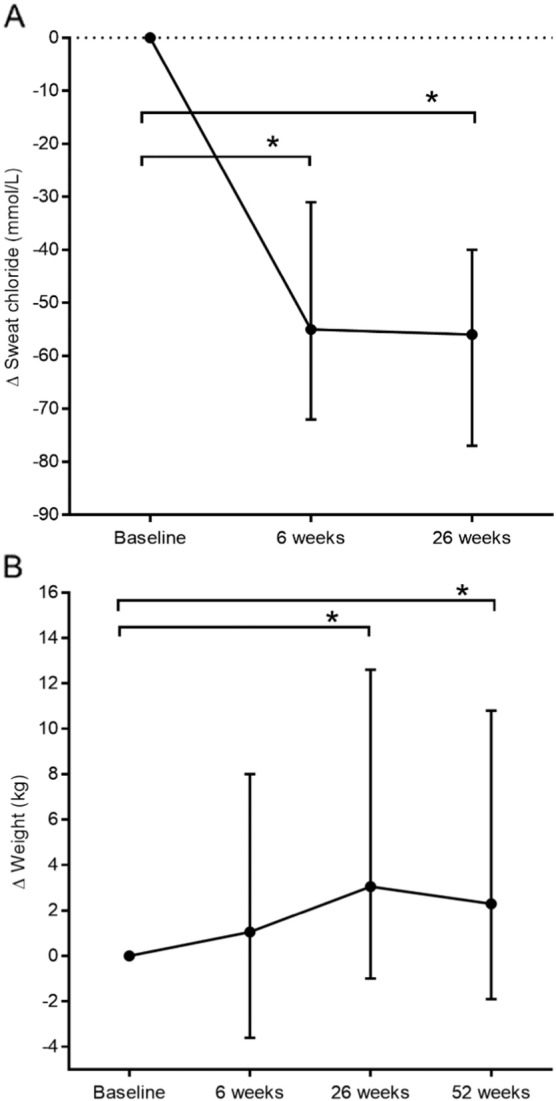
The clinical parameters analysed from baseline over a time frame of 52 weeks treatment with ivacaftor (150 mg bd) at a follow-up period of 6, 26 weeks and (omitting sweat chloride) 52 weeks. *P ≤ 0 ∙ 05. (A) The median sweat chloride (mmol/L) at baseline was 109 mmol/L (range 61 to 131). The absolute change was − 55 at 6 weeks (range − 72 to − 31; P = 0.0001) and − 56 at 26 weeks (range − 77 to − 40; P = 0.0001). (B) The baseline median weight (kg) was 64 kg (range 41 to 83), 63 kg at 6 weeks (range 41 to 87); increasing to 66 kg at 26 weeks (range 43 to 92; P = 0.001 versus baseline) and 65 kg at 52 weeks (range 41 to 90; P = 0.01 versus baseline).

The median sweat chloride concentration was 109 mmol/L (range 61 to 131) at baseline. The median significantly decreased to 51 mmol/L (range 27 to 94) at the six week follow-up (P = 0 ∙ 0005 versus baseline), the median change was − 55 mmol/L (range − 72 to − 31). There were two patients with a sweat chloride concentration falling below 39 mmol/L at the six week follow-up, into the normal range. The concentrations remained significantly decreased following 26 weeks of treatment at a median of 45 mmol/L (range 20 to 91; P < 0 ∙ 0005 versus baseline), the median change was − 56 (range − 77 to − 40). There were six patients with normal sweat chloride concentrations 26 weeks post ivacaftor. (Friedman test n = 12 P = < 0.0001) (Fig. 3A).

### 3.6 Weight

The median weight at baseline was 64 kg (range 41 to 83). After 6 weeks of treatment, the median weight was 63 kg (range 41 to 87). The median weight increased significantly at the 26 week follow-up by 3 kg (range − 1 to 13) from baseline, to a median of 66 kg (range 43 to 92; P = 0 ∙ 001 versus baseline). The weight remained significantly increased by 2 kg (range − 2 to 11) from baseline, to a median of 65 kg (range 41 to 90; P = 0 ∙ 01 versus baseline) at 52 weeks (Friedman test n = 12 P = < 0.02) (Fig. 3B).

## 4 Discussion

In this study we have shown that patients with CF and the G551D mutation undergoing treatment with ivacaftor experience a significant decline in symptoms of EOR. We also provided rare longitudinal data in a clinical, non-trial cohort, showing that ivacaftor treatment was associated with rapid changes in sweat chloride and clinically significant gains in lung function, as documented in clinical trials [8]. Significant weight gain was also seen following prolonged treatment of ivacaftor in our patient cohort. Interestingly, a small fall in measures of lung function and weight was seen 52 weeks, compared to 6 and 26 weeks. Such variation may be typical of real world data, and patients will often show some fluctuation of measurements in a complex clinical disease and longer term follow-up is warranted.

It is evident that ivacaftor treatment represents an opportunity to investigate clinically relevant aspects of CF pathophysiology in a patient setting characterised by increased CFTR function [2], [3], [4], [5], [6], [7], [8], [9]. Recent decades have seen improved success in managing patients with CF, with an increased life expectancy. We expect that knowledge regarding CF co-morbidities aside from pulmonary manifestations, is likely to become increasingly important [2].

Gastro-oesophageal reflux is a recognised co-morbidity in CF and occurs in up to 90% of patients [4]. We have previously shown that reflux and aspiration can be an important injury in patients with CF both in end stage disease [5] and following lung transplantation [13], [14]. It is therefore possible that reflux and aspiration could be a significant, chronic, and persistent contributor to CF pathophysiology [5], [7], [13], [14], [15]. It was of interest that patients who were experiencing symptoms of reflux in our study had worse lung function than those with lower levels of symptoms. We found that ivacaftor was beneficial for patients with symptoms of EOR, which is thought to be a precursor to microaspiration. Our findings may therefore be important both in terms of managing gastrointestinal symptoms associated with CF per se but also because reflux and aspiration may contribute to chronic lung disease in CF.

Evidence provided by a recent longitudinal cohort study involving the North American Cystic Fibrosis Therapeutics Development Network indicates a role of CFTR in gastrointestinal physiology following ivacaftor treatment [3]. The authors suggested that their observations provided “seminal information” regarding a possible mechanism underlying the clinical benefits of gastrointestinal disease following ivacaftor such as observed weight gain. A total of seven patients prescribed ivacaftor (150 mg bd) were obtained from three centres yielding telemetric data monitoring postprandial intestinal pH. The authors found that the duodenal pH levels of CF patients observed after 1 month of ivacaftor treatment were comparable to those seen in normal individuals [3]. In normal individuals, the pH of the intestines following gastric emptying is 7.0 to 8.5. This indicated that after 1 month of ivacaftor treatment there was a pronounced effect on duodenal alkalization after gastric emptying, attributed to CFTR potentiation. The novelty of such data was identified in an accompanying editorial which encouraged further study [2]. To our knowledge, the effects of ivacaftor on EOR symptoms have not been investigated and we feel that our study therefore supports and complements the findings of Rowe et al. [3] providing data from a European patient setting, with longer term follow-up of 52 weeks, and in a larger group of patients.

Our finding, that patients treated with PPIs were symptomatic for EOR prior to ivacaftor was of interest to us. As reflected in our data there is frequent administration of PPI's in patients with CF. This is due to the fact the prevalence of reflux is high, and to optimise the effectiveness of PERT in CF. The use of PPIs has recently been associated with a dysregulated gastric microbiome in paediatric studies and links suggested between reflux, aspiration and the lung microbiome [16], [17]. Our data could be consistent with a situation where PPI usage does not prevent EOR but may also be associated with abnormal bacterial growth in the stomach with iatrogenic potential. We suggest that further research is indicated in this regard, which may be especially relevant in CF.

Our study has limitations including its observational nature and lack of placebo control. Arguably, our patient reported outcomes may be biased by non-specific treatment benefit such as effects of improved lung function. A strength of our study is that it provides new information about reflux in CF in a very rare setting. We recruited 5% of the CF population attending the Regional CF clinic in Newcastle upon Tyne, this percentage reflects the G551D population in the United Kingdom [18], however a larger group would have increased statistical power. Furthermore, our study provided an extended longitudinal follow-up of 12 months. The collection of data within a real world setting has been advocated [2], [19] to complement the evidence base from high quality clinical trials [8]. This small study provides initial real world data which might prompt further research which might include invasive physiological testing such as pH impedance studies. We would suggest that future studies include biomarker and physiological end points, integrated with patient reported outcomes. Such studies may provide increased insights into a multi-system pathophysiology and potential therapeutic approaches, in an exciting new era of precision medicine in CF.

## Funding

This work was supported by the BBSRC [grant number BB/F01/5895/1].

C Ward, J Pearson and Rhys Jones were supported by a UK Government Technology Strategy Board Knowledge Transfer Partnership (Certificate KTP008821). M Brodlie was supported by a Medical Research Council Clinician Scientist fellowship (MR/M008797/1).

## Conflict of interest

There were no conflicts of interest.

## Authors' contributions

All authors are credited for meeting the ICMJE authors contribution criteria described as:

1. Substantial contributions to the conception or design of the work; or the acquisition, analysis, or interpretation of data for the work; and
2. Drafting the work or revising it critically for important intellectual content; and
3. Final approval of the version to be published; and
4. Agreement to be accountable for all aspects of the work in ensuring that questions related to the accuracy or integrity of any part of the work are appropriately investigated and resolved.

Gemma Zeybel was responsible for the design of the study; patient recruitment and sampling. She analysed the data and co-authored the manuscript.

Amaran Krishnan was a clinical gastrointestinal investigator who contributed to study design. He reviewed and contributed to manuscript drafts.

Stephen Bourke, Simon Doe and Alan Anderson were responsible for the patient recruitment, sample collection and phenotyping of the CF patients. They contributed to and critically reviewed the manuscript.

Shoaib Faruqi and Alyn H Morice contributed to the study design, interpretation of the data and providing a critical review of the manuscript. Peter W Dettmar contributed to the conception of the work, supervision and manuscript review.

Rhys Jones and Mellissa McDonnell contributed to the study design, in particular, they performed a literature review of reflux questionnaires to determine our study tools.

Mujdat Zeybel was a Marie Curie fellow who contributed to the data interpretation and analysis. He also critically reviewed the manuscript.

Malcolm Brodlie was a clinician scientist CF specialist who contributed to the study design and manuscript writing, he also contributed to the role of lead investigator.

Jeffrey Pearson, and Chris Ward were lead investigators, responsible for the study design and interpretation of the data. Jeffrey Pearson critically reviewed the manuscript. Chris Ward wrote the manuscript drafts with Gemma Zeybel.

## Figures and Tables

**Table 1:** Demographics and characteristics of subjects at baseline. Note: FEV_1_ forced expiratory volume in 1 s; FVC forced vital capacity; PS pancreatic sufficiency; PPI Proton Pump Inhibitor; H2RA Histamine 2 receptor agonist; – no PPI or H2RA; NMD no mutation detected.

Subject	Sex	Age (years)	PS	Acid suppression	Genotype
1	F	20	Yes	PPI	G551D/P67L
2	M	22	No	PPI	G551D/F508del
3	F	21	No	–	G551D/G542X
4	F	21	No	–	G551D/F508del
5	F	17	No	PPI	G551D/F508del
6	F	38	No	H_2_RA	G551D/F508del
7	F	20	No	PPI	G551D/F508del
8	M	23	No	PPI	G551D/F508del
9	M	25	No	H_2_RA	G551D/F508del
10	M	28	No	PPI	G551D/F508del
11	M	24	No	PPI	G551D/F508del
12	M	28	No	–	G551D/W401X
Median (range)		24 17–38			
